# The link between intraneuronal N-truncated amyloid-β peptide and oxidatively modified lipids in idiopathic autism and dup(15q11.2-q13)/autism

**DOI:** 10.1186/2051-5960-1-61

**Published:** 2013-09-16

**Authors:** Janusz Frackowiak, Bozena Mazur-Kolecka, N Carolyn Schanen, W Ted Brown, Jerzy Wegiel

**Affiliations:** 1 Department of Developmental Neurobiology, NYS Institute for Basic Research in Developmental Disabilities, New York, Staten Island, USA; 2 Nemours Biomedical Research, duPont Hospital for Children, Wilmington, DE, USA; 3 Department of Human Genetics, NYS Institute for Basic Research in Developmental Disabilities, New York, Staten Island, USA

**Keywords:** Idiopathic autism, Chromosome-15q11.2-q13 duplication, Amyloid-β peptide, Oxidative stress, Malondialdehyde, 4-hydroxy-2-nonenal

## Abstract

**Background:**

Autism is a neurodevelopmental disorder of unknown etiopathogenesis associated with structural and functional abnormalities of neurons and increased formation of reactive oxygen species. Our previous study revealed enhanced accumulation of amino-terminally truncated amyloid-β (Aβ) in brain neurons and glia in children and adults with autism. Verification of the hypothesis that intraneuronal Aβ may cause oxidative stress was the aim of this study.

**Results:**

The relationships between neuronal Aβ and oxidative stress markers—4-hydroxy-2-nonenal (HNE) and malondialdehyde (MDA)—were examined in the frontal cortex from individuals aged 7–32 years with idiopathic autism or with chromosome 15q11.2-q13 duplications (dup(15)) with autism, and age-matched controls. Quantification of confocal microscopy images revealed significantly higher levels of neuronal N-truncated Aβ and HNE and MDA in idiopathic autism and dup(15)/autism than in controls. Lipid peroxidation products were detected in all mitochondria and lipofuscin deposits, in numerous autophagic vacuoles and lysosomes, and in less than 5% of synapses. Neuronal Aβ was co-localized with HNE and MDA, and increased Aβ levels correlated with higher levels of HNE and MDA.

**Conclusions:**

The results suggest a self-enhancing pathological process in autism that is initiated by intraneuronal deposition of N-truncated Aβ in childhood. The cascade of events includes altered APP metabolism and abnormal intracellular accumulation of N-terminally truncated Aβ which is a source of reactive oxygen species, which in turn increase the formation of lipid peroxidation products. The latter enhance Aβ deposition and sustain the cascade of changes contributing to metabolic and functional impairments of neurons in autism of an unknown etiology and caused by chromosome 15q11.2-q13 duplication.

## Background

Autism is a complex neurodevelopmental disorder characterized by impaired reciprocal social interactions, impaired verbal and nonverbal communication, and stereotyped patterns of repetitive behavior, with clinical symptoms developing in early childhood [1]. The cause of autism is unknown, but a combination of genetic, epigenetic, and environmental factors has been proposed to play a major role in the etiology of autism. Certain genetic disorders are associated with a particularly high incidence of autism, e.g., it is as high as 69% among individuals who have a duplication of chromosome 15q11.2-q13 of maternal origin [dup(15)] [2,3].

Altered metabolism of amyloid precursor protein (APP) in autism is indicated by higher plasma levels of secreted APP—two or more times—in children with severe autism and aggression than in children without autism, and by lower levels of amyloid-beta (Aβ) 40 than in controls [4,5]. Hence, an increased processing of APP by alpha-secretases has been proposed to contribute to autism [5,6]. Our recent postmortem studies demonstrated intracellular deposits of Aβ truncated on the amino-terminal side—the Aβ17-40/42—in neurons and glia in the brain cortex, cerebellum and in subcortical nuclei. The percentage of amyloid-positive neurons as well as intraneuronal Aβ load were significantly higher in individuals diagnosed with idiopathic autism and in subjects diagnosed with dup(15) and autism spectrum disorder (ASD) than in controls [7]. The pathological consequences of intracellular accumulation of N-truncated Aβ by neurons and glia are not known. However, accumulation of full-length Aβ 1-42 and Aβ 1-40 in Alzheimer’s disease and Down syndrome is associated with production of reactive oxygen species and contributes to oxidative stress [8-10].

Increased formation of reactive oxygen species has been implicated in the pathophysiology of autism, and markers of oxidative stress have been detected in autism, even at an early age. Peripheral blood in children with autism contains elevated levels of malondialdehyde (MDA), an indicator of lipid peroxidation [11], and increased levels of thiobarbituric acid–reactive substances (TBARS) [12]. Increased oxidative stress in children with autism was also suggested by increased nitric oxide (NO) levels in red blood cells [13] and higher urinary excretion of TBARS, lipid hydroperoxides, 4-hydroxy-2-nonenal (HNE), and protein carbonyls along with low levels of urine antioxidants [14]. Severity of autism appears to be correlated with urinary excretion of 8-isoprostane-F2 alpha [15]. A link of oxidative stress in autism to malfunction of anti-oxidative mechanisms is indicated by reduced serum levels of ceruloplasmin and transferrin—the major proteins with anti-oxidative properties [11], as well as significantly lower ratio of reduced/oxidized glutathione in the plasma, decreased methionine cycle turnover [16,17], increased plasma activities of glutathione peroxidase [13], xanthine oxidase and superoxide dismutase (SOD) [12], and decreased catalase activity [12].

Oxidative stress in the brain in autism is indicated by oxidative DNA damage: increased levels of 3-nitrotyrosine (3-NT) [18-20] and 8-oxo-deoxyguanosine [18]. The distribution of 3-NT indicates brain region–specific enhancement of oxidative stress, particularly in cerebellum and cortical areas involved in speech processing, sensory and motor coordination, emotional and social behaviors, and memory [20]. Other biomarkers of oxidative stress—reduced glutathione (GSH) levels, and decreased ratio of GSH to oxidized glutathione in cerebellum and temporal cortex—indicate a role of deficient glutathione antioxidant defense in certain brain regions in the development of oxidative stress in autism [18,21]. The pathomechanisms of oxidative stress in autism have not been determined; however, it can be attributed, in part, to activation of the immune system, which has been confirmed in the brain [22] and in the peripheral blood [23]. Significantly higher levels of 3-chlorotyrosine—a biomarker of a chronic inflammatory response—in cerebellum and temporal cortex may link oxidative stress in the brain in autism with a chronic inflammatory response, whereas a decreased aconitase activity in the cerebellum in autism indicates increased mitochondrial superoxide production [18].

The aim of this project was to test the hypothesis that intracellular deposits of N-truncated Aβ are not biologically neutral, but may be a source of reactive oxygen species in brain cortex neurons in subjects with idiopathic autism and in subjects with dup(15) with autism.

## Methods

### Brain tissue

Samples of autopsy brain frontal cortex, formalin-fixed and frozen, were obtained from the Brain Bank and Tissue Bank for Developmental Disabilities and Aging, of the New York State Institute for Basic Research in Developmental Disabilities Staten Island, NY, the Harvard Brain Tissue Resource Center, Belmont, MA, and the NICHD Brain and Tissue Bank for Developmental Disorders, University of Maryland School of Medicine, Baltimore, MD (Tables 1 and 2). Selection of this brain region was based on neuropathological changes detected in autism [24]. Idiopathic autism was confirmed by Autism Diagnostic Interview–Revised (ADI-R) score. Duplication of 15q11.2-q13 was confirmed by genotyping with 19–33 short tandem repeat polymorphisms from chromosome 15, custom and/or array comparative genomic hybridization (array CGH), Southern blot analysis of dosage with 5–12 probes, and fluorescent in situ hybridization performed using antemortem peripheral blood samples and lymphoblast cell lines [2,3].

**Table 1:** Formalin-fixed brains tested

**Group**	**Brain bank number**	**Sex**	**Age years**	**Cause of death**	**PMI**
dup(15)	B-7359	M	9	Cardiac arrest	13.6
dup(15)	B-7741	M	10	SUDEP	17.7
dup(15)	B-7014	M	11	Seizure-related	10.5
dup(15)	B-6973	F	15	SUDEP	24
dup(15)	B-7619	F	15	Aspiration pneumonia	-
dup(15)	B-7041	M	20	Cardiac arrest, resusc./ventilation	28
dup(15)	B-7436	M	24	Seizure-related	36
Autism	HSB4640	M	8	Asthma attack	13.8
Autism	B-6349	M	9	Cardiopulmonary arrest	3.8
Autism	CAL105	M	11	drowning	-
Autism	CNL93-01	M	23	Seizure related	14
Autism	B-6994	M	28	Seizure-related	43
Autism	NP06-54	M	32	Brain tumor	-
Control	UMB1706	F	8	Rejection of cardiac transplant	20
Control	UMB1670	M	14	Asphyxia (hanging)	5
Control	UMB4722	M	14	Multiple traumatic injuries	16
Control	BTB3960	F	25	Not known	26
Control	CNL291-00	M	32	Heart failure	14
Control	CNL212-98	F	33	bronchopneumonia	16

NOTE: postmortem interval (PMI); Sudden unexpected and unexplained death of subject with known epilepsy (SUDEP).

**Table 2:** Frozen brains tested

**Group**	**Brain bank number**	**Sex**	**Age years**	**Cause of death**	**PMI**
dup(15)	B-7359	M	9	cardiac arrest in seizures	13.6
dup(15)	B-7014	M	11	seizure-related	10.5
dup(15)	B-7041	M	20	Cardiac arrest, resusc./ventilation	28
dup(15)	B-6856	F	26	asphyxia, seizure suspected	28.6
Autism	UMB-5144	M	7	complications of tumor	3
Autism	UMB-4849	M	7	drowning	20
Autism	CNL-1480	M	8	asthma	13.8
Autism	UMB-4721	M	8	drowning	16
Autism	B-5342	F	11	drowning during seizures	13
Autism	UMB-4899	M	14	drowning	9
Autism	UMB-5278	F	16	drowning in seizure disorder	13
Autism	UMB-5176	M	22	SUDEP	18
Autism	B-6640	F	29	seizure disorder	18
Control	UMB-4898	M	7	drowning	12
Control	UMB-4337	M	8	neck injury	16
Control	UMB-5391	M	8	drowning	12
Control	UMB-5161	F	10	accident-hanging	22
Control	UMB-5334	M	12	suicide-hanging	15
Control	CNL-1398	M	13	asphyxia-hanging	5
Control	UMB-4722	M	14	multiple injuries	16
Control	UMB-5163	M	15	drowning	12
Control	UMB-5168	F	16	cardiac arrhythmia	11
Control	B-5251	M	19	pneumonia	18.6
Control	UMB-4590	M	20	cardiac arrest	19
Control	B-5718	M	22	N/A	21.5
Control	UMB-818	M	27	accident - multiple injuries	10
Control	CNL-247	M	31	N/A	3
Control	CNL-1169	M	32	congestive heart failure	14

NOTE: postmortem interval (PMI); not assigned (N/A); Sudden unexpected and unexplained death of subject with known epilepsy (SUDEP).

### Immunofluorescence and confocal microscopy

Free-floating 50-μm sections of formalin-fixed and polyethylene glycol–embedded frontal cortex containing Brodmann cortical areas 9 and 46 were used for detection of Aβ, lipid peroxidation products, and markers of cell organelles by immunofluorescence and confocal microscopy. The antibodies used are listed in Table 3. Secondary antibodies were affinity-purified donkey antisera labeled with Alexa 488 or 555 specific for the respective species (Invitrogen/Molecular Probes, Grand Island, NY, USA). Nuclei were counterstained with TO-PRO-3-iodide (TOPRO-3i) (Invitrogen/Molecular Probes). Images were collected using a Nikon C1 confocal microscope system and with EZC1 image analysis software. The images were used for further immunofluorescence quantification, basing on previous studies which have shown that measurements of immunofluorescence staining allow relative protein quantification in tissue sections when properly standardized methods are used [25]. The guidelines for proper image acquisition and controlling factors that affect the accuracy and precision of quantitative fluorescence microscopy were applied [26]. To ensure unbiased sampling of images for measurements of fluorescence intensity, sections were coded and microscopic fields in the 3^rd^ and 5^th^ cortical layers were randomly selected in the blue channel in which only cell nuclei and cytoplasm were visible, and large pyramidal neurons were identified by their morphology. For measurements, confocal image layers containing cytoplasm and nucleus of pyramidal neurons were selected. Images in three channels were collected at the amplifications at which the background staining was minimal, and the settings of channel amplification were the same for all groups tested. Specificity of immunostainings was confirmed as previously described [27-29]. The background autofluorescence for pyramidal neurons and neuropil was measured in unstained sections and in sections stained with omission of primary antibodies. Specific immuofluorescence was calculated after subtracting autofluorescence and nonspecific background fluorescence. The levels of immunofluorescence intensities for Aβ, HNE, and MDA were measured using Image J software (NIH), and specific immunostainings were calculated per 1,000 pixels of cell contour or random samples of surrounding neuropil of the pixel size comparable to neurons. For each brain and each immunostaining an average of 38 cells were measured. Blood vessels were excluded from measurements.

**Table 3:** Antibodies used for immunohistochemistry and for immunoblotting

**Name**	**Epitope or target**	**Dilution**	**Host/type**	**Source**
6E10	4-13 aa Aβ	1:2,000	M-monocl	Signet Laboratories (antibody developed at IBRDD [30,31])
4G8	17-21 aa Aβ	1:2,000	M-monocl	IBRDD [30,32]
R226	36-42	1:40	R-polycl.	IBRDD [33]
Anti-MDA	malondialdehyde	1:500	R-polycl	Alpha Diagnostic Int., San Antonio, TX
			G-polycl	
Anti-HNE	4-hydroxy-2-nonenal	1:500	R-polycl	Alpha Diagnostic Int., San Antonio, TX
COX IV	Mitochondria	1:100	R-monocl	Cell Signaling Technology
LAMP 1	Lysosomes	1:400	R-polycl	Abgent
actin	actin	1:4000	M-monocl	Pierce/Thermo Sci., Rockford, IL
LC3B	Autophagic vacuoles	1:100	R-monocl	Cell Signaling Technology Danvers, MA
8C4	Tripeptidyl peptidase I	1:30	M-monocl	IBRDD [34]
GFAP	Astrocytes	1:400	R-polycl	Sigma, St. Louis, MO
SP15	synaptophysin	1:100	M-monocl	Calbiochem-EMD Biosciences, Inc., La Jolla, CA
synaptophysin	synaptophysin	1:200	G-polycl	GeneTex, Irvine, CA

Antibodies were monoclonal or polyclonal: Mouse (M); Rabbit (R) or Goat (G).

### Immunoblotting

Samples of frozen frontal cortex were homogenized in glass-teflon homogenizer in 10 mM TRIS buffer, pH 7.5, containing 0.15 M NaCl, 0.65% NP-40, and protease inhibitor cocktail (Roche Diagn. GmbH, Mannheim, Germany), and blood vessels and leptomeninges were removed by passing through 75 μm nylon mesh. Protein contents were measured using the BCA protein assay (Pierce, Rockford, IL). Samples of brain lysates were centrifuged at 10,000 g for 10 min, and supernatants and pellets were collected. Samples containing 20 μg of protein lysate as well as supernatants and pellets obtained from 20-μg samples of full lysate were subjected to SDS-PAGE (8% tris/tricine gels). Full lysates (10 μg protein samples) were used for slot blotting. Proteins modified by MDA and HNE were detected with the antibodies listed in Table 3. The levels of actin were used as sample load controls. Densitometrical measurements of bands on membranes were performed with SigmaGel software (Jandel Sci., San Raphael, CA, USA).

### Statistical analysis

For all data groups the degrees of asymmetry of the data distribution around mean values have been calculated. Because the data did not have normal distribution (measured as skewness of the distribution) natural logarithms of values were used for the Student’s t-test analysis. To test specific hypotheses, pairwise comparisons were calculated using Student’s t-test adjusted for the non-homogeneity of variance between two groups.

## Results

### Immunohistochemical reactions for Aβ

**Figure 1:**
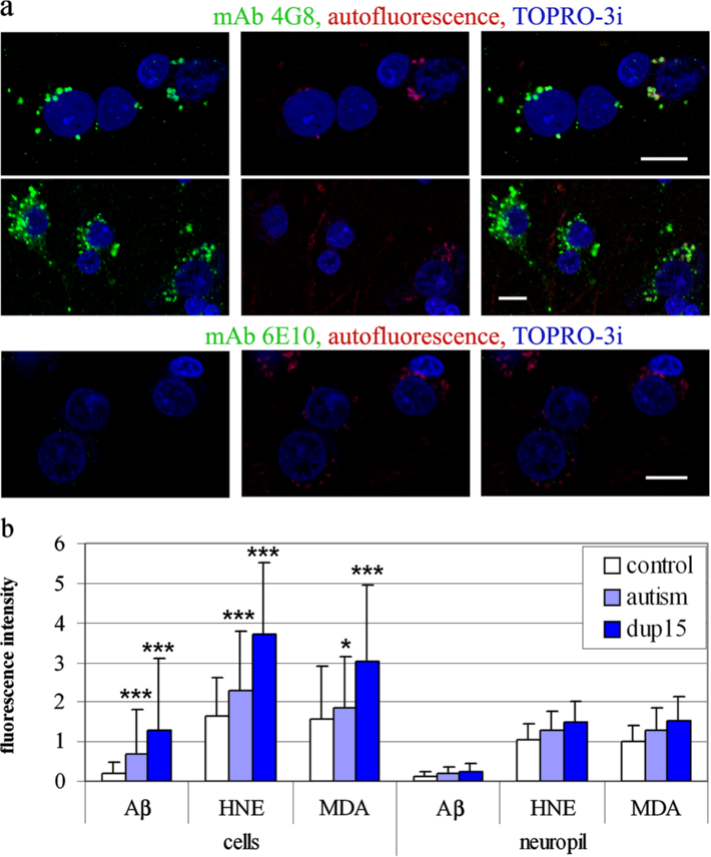
**N-terminally truncated Aβ in pyramidal neurons in frontal cortex in confocal microscopy and semi-quantification of N-truncated Aβ, HNE and MDA in neurons and in neuropil. (a)** Large pyramidal neurons in frontal cortex in layers 3 and 5 in a 10-year-old individual with dup(15)/autism contain granules immunoreactive with mAb 4G8, but not with 6E10, consistent with N-terminally truncated Aβ. Between 20% and 40% of intracellular granular Aβ in this brain are co-localized with autofluorescent granules of lipofuscin. *Bars* 10 μm. **(b)** Intensities of the Aβ, MDA and HNE immunoreactivities in individual neurons in the frontal cortex for all brains listed in Table 1 are significantly larger in the dup(15)/autism cases and in the idiopathic autism than in controls. The bars show average + SD. Statistical significance: * p < 0.05, *** p < 0.001.

Aβ immunoreactivity was detected in neurons, glia, and in neuropil by immunofluorescence and confocal microscopy in all the control, idiopathic autism, and dup(15)/autism brain tissue samples. The intraneuronal Aβ deposits immunoreacted with two antibodies against distinct Aβ epitopes: mAb 4G8, specific for the 17-21 aminoacids of the Aβ sequence (Figure 1a) and R226, specific for aa 36-42 of Aβ (not shown) but revealed almost no reaction with mAb 6E10, specific for aa 4-13 of Aβ (Figure 1a), indicating that the deposits contained N-terminally truncated Aβ, consistent with Aβ 17-40/42 or Aβ 11-40/42 (the products of secretase-α and –γ, or secretase-β and –γ, respectively), or other Aβ species. The N-truncated Aβ was detected in up to 60% of large pyramidal neurons in the frontal cortex in the dup(15)/autistic subjects, and in 30– 45% of neurons in idiopathic autism. Control brains contained a weaker immunoreactivity for N-truncated Aβ, which was detected in 15–35% of neurons. The intracellular Aβ-immunoreactive granules were of diameter 0.3–2.5 μm, and their numbers in individual large pyramidal neurons in layers 3 and 5 varied greatly in each case in every group tested, from no reaction or a few granules, to multiple granules filling the pericaryon (Figure 1a). The morphology of nuclei in cells without and with Aβ deposits was similar, and chromatin did not show changes typical for apoptosis. Between 10% and 50% of intraneuronal deposits of Aβ were co-localized with autofluorescent material consistent with lipofuscin granules (Figure 1a).

To quantify the intensities of immunostaining, the specific immunofluorescence, i.e., immunofluorescence after subtraction of nonspecific background fluorescence and autofluorescence, was measured for cells and neuropil. The average intensities of specific reactions for Aβ measured in pyramidal neurons in the confocal image layer containing cytoplasm and nucleus were significantly higher in dup(15)/autism and in idiopathic autism than in controls (p < 0.001). The reactions in dup(15)/autism were significantly more intense than in idiopathic autism (p < 0.01) (Figure 1b, Aβ bars). The intensities of the immunoreactions for Aβ in the neuropil did not differ between the groups studied.

### Lipid peroxidation products

**Figure 2:**
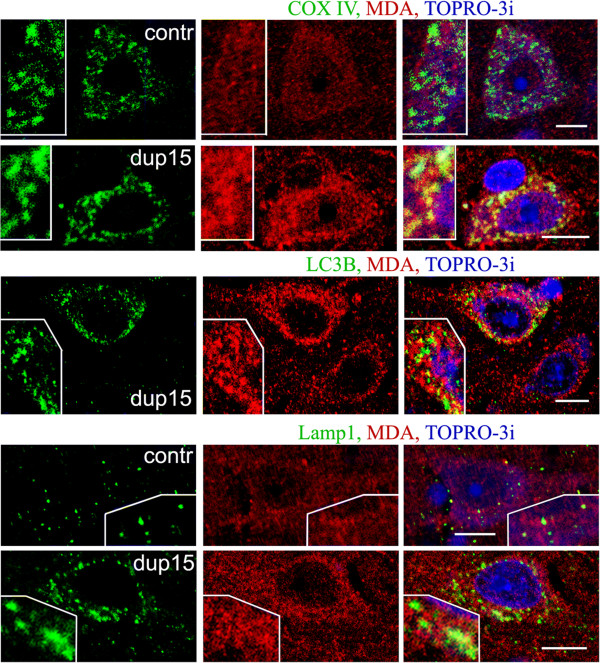
**MDA immunoreactivity in large pyramidal neurons in layers 3 or 5 of frontal cortex in 10-year-old individual with dup(15)/autism [dup(15)] and in 8-year-old control subject (contr) brains.** Mitochondria, visualized by immunostaining for cytochrome c oxidase COX IV, were the site of weak reactivity for MDA in control but a strong reactivity in dup(15)/autism brain. Most autophagic vacuoles (detected with antibody LC3B) and lysosomes/late endosomes (detected by the presence of Lamp-1 glycoprotein) did not contain a significant fraction of MDA. *Bars* 10 μm.

Immunoreactivities for HNE and for MDA were detected in all layers of the frontal cortex in all the brains examined that were diagnosed with idiopathic autism or dup(15)/autism and in controls. The immunoreactions for HNE and for MDA were detected in granules with diameter 0.25–3.5 μm, located in the cytoplasm of neurons and glia, in the neuropil, and in blood vessel walls. To characterize the subcellular localization of lipid peroxidation products in large pyramidal neurons in layers 3 and 5, their presence in mitochondria, autophagic vacuoles, lysosomes, and lipofuscin was verified by confocal microscopy. Mitochondria were visualized with an antibody against cytochrome c oxidase, COX IV. The intraneuronal granules that were most intensely immunoreactive for HNE or MDA were frequently identified as COX IV–positive mitochondria in dup(15)/autism (Figure 2) and in idiopathic autism (not shown). However, the majority of neuronal granular immunoreaction for lipid peroxidation products was not associated with mitochondria. Mitochondria in control brains contained lipid peroxidation products but the immunoreactions were much weaker than in dup(15) and autism (Figure 2).

Autophagic vacuoles were detected with an antibody against the LC3B marker. Between 30% and 60% of LC3B-positive granules were immunoreactive for MDA (Figure 2) and HNE (not shown) but these organelles contained no more than 5–10% of all granules immunoreactive for lipid peroxidation products in neurons.

**Figure 3:**
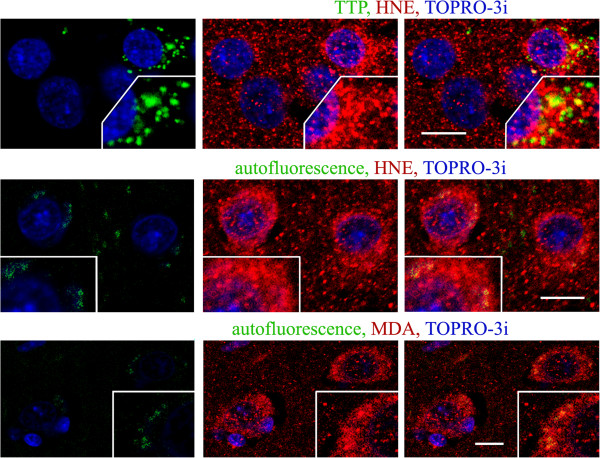
**Large pyramidal neurons in layer 5 of frontal cortex in 10-year-old individual with dup(15)/autism.** Lysosomes identified by the presence of tripeptidyl peptidase I (TPP), characteristic for activated cells (using mAb 8C4), were detected in cells with more intense immunoreactivity for HNE. Up to 50% of these lysosomes immunoreacted strongly for HNE. Lipofuscin, detected as autofluorescent granules in green channel, contained strong reactivities for HNE and MDA, but most lipid peroxidation products were not located in lipofuscin. *Bars* 10 μm.

Lysosomes and late endosomes, detected by the presence of Lamp-1 glycoprotein, contained granules reactive for HNE and MDA, but only about 30% of lysosomes/enodomes were the site of prominent accumulation of lipid peroxidation products in autism, dup(15)/autism and controls (Figure 2). Among the lysosomes identified by the presence of tripeptidyl peptidase I—a lysosomal peptidase with a broad substrate specificity—up to 50% contained a significant or strong reaction for lipid peroxidation products (Figure 3, TTP).

Between 30% and 50% of pyramidal neurons in autism and in dup(15)/autism contained intracellular granules that revealed a wide-spectrum autofluorescence detected in the red-orange, green, and deep red channels—consistent with the properties of lipofuscin. The number of autofluorescent lipofuscin granules varied from a single profile to over 20 in a single cell image. They all were immunoreactive for HNE and MDA; however, only a minority of all neuronal immunoreactivities for both lipid peroxidation products was associated with autofluorescent lipofuscin (Figure 3).

**Figure 4:**
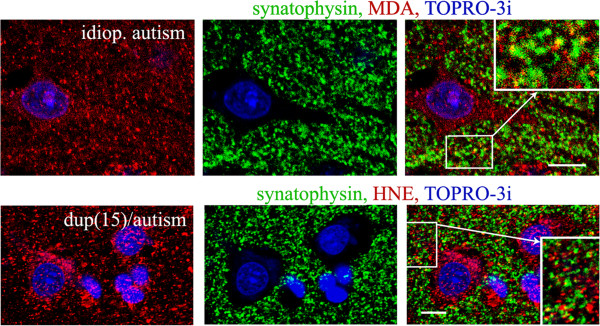
**HNE and MDA in 23-year-old individual with idiopathic autism and in 10-year-old individual with dup(15)/autism.** Up to 5% of synaptic terminals (detected by the presence of synaptophysin) contained lipid peroxidation products. *Bars* 10 μm.

Nuclear immunoreactions for HNE and MDA in large pyramidal neurons showed a significant variability between the cases and between individual neurons in each brain. Each brain that was tested contained large pyramidal neurons in the frontal cortex with intense as well as with scanty immunoreactions (Figures 2, 3, 4).

Granular reactions for HNE and MDA were also located in the neuropil. Up to 15% of these HNE- and MDA-reactive profiles were co-localized with synapses, as indicated by double staining for synaptophysin (Figure 4). The percentage of synapses that contained granules immunoreactive for MDA or HNE was 1.5–5% in dup(15)/autism and idiopathic autism and control.

Measurements of intensities of specific immunoreactivity for HNE and MDA detected in individual pyramidal neurons in the confocal image layers containing cytoplasm and nucleus revealed significantly higher average levels in dup(15)/autism and in autism than in controls (p < 0.001). The signals in the dup(15)/autism samples were significantly more intense than in idiopathic autism (p < 0.01). The immunoreactions for lipid peroxidation products in the neuropil were similar in the groups studied (Figure 1b: HNE and MDA bars).

**Figure 5:**
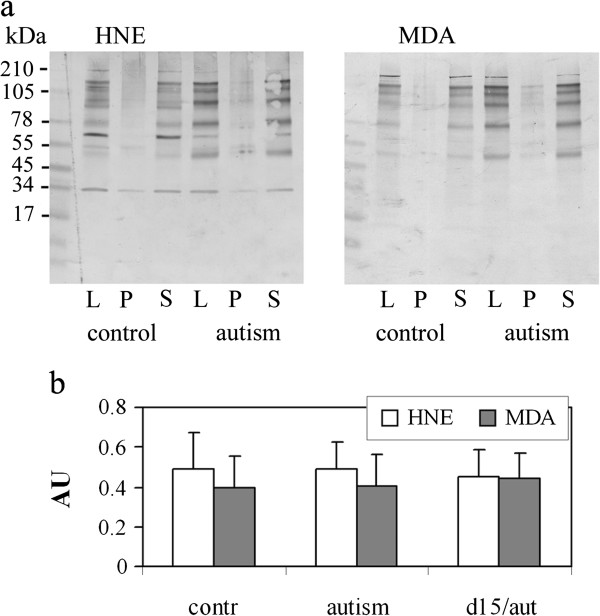
**HNE- and MDA-modified proteins in brain lysates. (a)** HNE- and MDA-modified proteins detected by immunoblotting in frontal cortex lysates of autism and control brains (lanes L). More than 85% of the modified proteins were detected in supernatant (S) after centrifugation at 10,000 g for 10 min, but not in the pellet (P). **(b)** The levels of total MDA- and HNE-modified proteins in lysates normalized to actin levels, as detected by slot blotting in all brains listed in Table 2, were similar in dup(15)/autism, idiopathic autism and in controls.

Proteins modified with HNE and MDA revealed by immunoblotting were of the molecular sizes 50–180 kD in samples from autistic and control subjects. More than 85% of the modified proteins were soluble in low concentrations of detergent—0.65% Nonidet NP-40 (Figure 5). There was a significant variability regarding the molecular size and intensity of the HNE- and MDA-modified protein bands detected in lysates, 10,000 g pellets or supernatants in the control and autism groups, and none of these HNE- and MDA-modified proteins was specific for autism. The levels of total MDA- and HNE-modified proteins detected in slot blots were similar in dup(15)/autism, idiopathic autism and in controls (Figure 5).

### The relationship between localization and levels of Aβ and lipid peroxidation products

**Figure 6:**
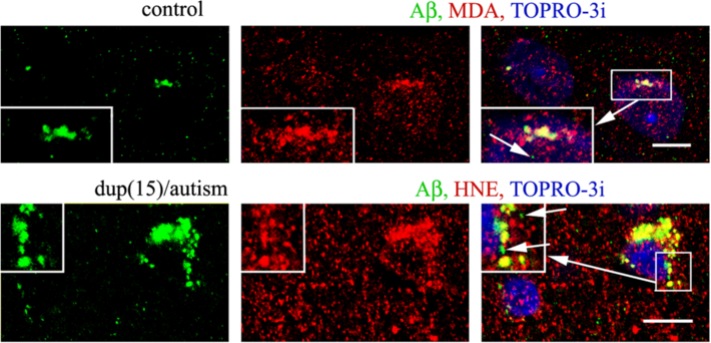
**Co-localization of Aβ and HNE and MDA.** Intracellular products of lipid peroxidation—HNE and MDA—appear as granules with diameter 0.25–3.5 μm. Aβ is co-localized with intracellular HNE products with the notable exception of the smallest Aβ granules, typically with diameter less than 0.3 μm, but infrequently also larger—up to 1 μm, as shown in dup(15)/autism and control brains. *Bars* 10 μm.

Double immunostaining and confocal microscopy revealed that the intracellular Aβ was almost entirely co-localized with HNE and MDA in all groups, with a notable exception of the smallest granules, typically of diameter up to 0.3 μm but infrequently reaching the diameter of 1 μm, which did not reveal reactions for lipid peroxidation products (Figure 6).

**Figure 7:**
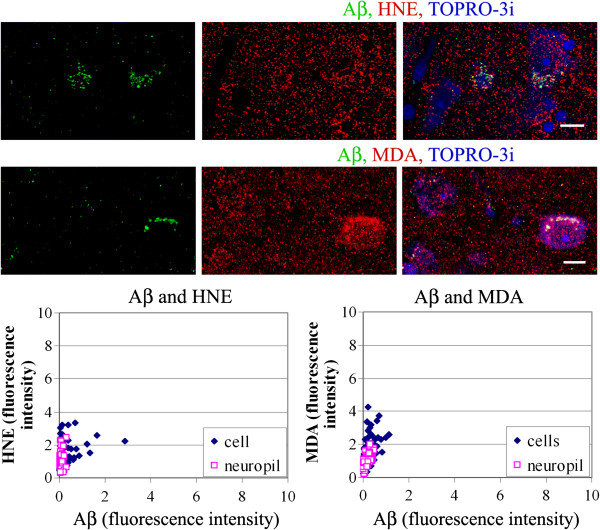
**Brains of control individuals, 8 years old and 25 years old contain intracellular granular immunoreactions HNE and MDA, the intensities of which are correlated with the amounts of accumulated Aβ, as shown in the confocal images.***Bars* 10 μm. The graphs show the relationship between Aβ and HNE or MDA for neurons in all control cases listed in Table 1.

The specific immunofluorescence for Aβ and for HNE and MDA was measured in individual neurons and in neuropil. Neurons with cytoplasmic Aβ granules contained granules reactive for HNE and MDA that were more numerous and more intensely stained as compared to cells that were Aβ-negative as well as to the surrounding neuropil. This relationship was observed in all groups studied. In control brains, the granular reactions for HNE and MDA were in the majority of neurons with similar intensities and distribution as in the surrounding neuropil, and stronger immunoreactions for HNE and for MDA were detected only in neurons immunoreactive for Aβ (Figure 7).

**Figure 8:**
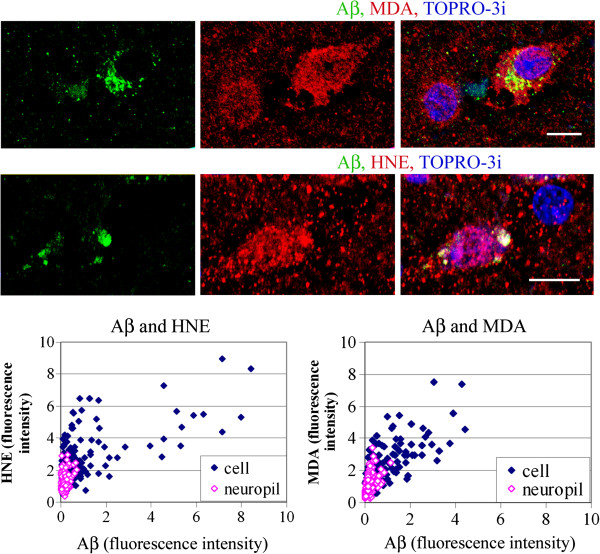
**Brains of individuals diagnosed with idiopathic autism, 23 and 24 years old.** The HNE and MDA intracellular immunoreactions are co-localized with Aβ, as shown in the confocal images, and their intensities are correlated with the amounts of accumulated Aβ. *Bars* 10 μm. The graphs demonstrate the correlation between the intensities of the immunoreactions measured for Aβ and HNE and MDA in all autism cases listed in Table 1.

**Figure 9:**
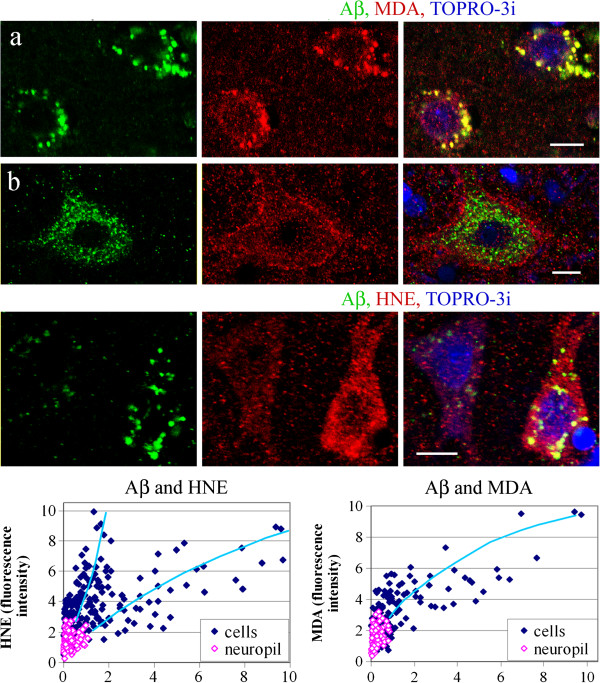
**Brains of individuals diagnosed with dup(15)/autism, 10 and 11 years old.** Confocal microscopy revealed Aβ in more than 50% of frontal cortex neurons in the dup(15) with autism. The immunoreactivity for HNE is granular and is located in neurons, glia and neuropil. Intracellular deposits of Aβ are the sites of strong accumulation of MDA **(a)**, but in one case of dup(15)/autism **(b)**, large pyramidal neurons in layers 3 and 5 in the cortex accumulating Aβ contain a particularly strong immunoreaction for MDA in the vicinity of plasma membrane. The reactions for lipid peroxidation products are correlated with cellular immunoreactivity for Aβ. *Bars* 10 μm. Measurements of the intensities of immunoreactions for Aβ and HNE in all dup(15) cases listed in Table 1 shown in the graph reveal two populations of neurons with distinct intracellular Aβ/HNE ratios.

The neuronal reactions for HNE and MDA in idiopathic autism were stronger than in control brains (Figure 1b, cells: HNE, MDA). The intensities of the HNE and MDA immunoreactions measured in individual cells were positively correlated with the amounts of Aβ immunoreactivity: the Pearson correlation coefficient values were r = 0.64 (p < 0.001) and r = 0.74 (p < 0.001), respectively (Figure 8). In dup(15)/autism the neuronal reactions for MDA were stronger than in control brains and in idiopathic autism (Figure 1b, cells: HNE, MDA). The intensities of the immunoreactions for MDA measured in individual cells were correlated with the amounts of Aβ immunoreactivity (r = 0.76, p < 0.001) (Figure 9). In one 11- year-old individual with dup(15)/autism, large pyramidal neurons in layers 3 and 5 with intracellular Aβ contained a strong immunoreaction for MDA close to the neuronal plasma membrane (Figure 9b). Double immunostaining for Aβ and HNE in dup(15)/autism revealed stronger neuronal HNE reactions than in control brains and in idiopathic autism (Figure 1b, cells: HNE). Two populations of neurons of similar numerical densities were revealed among cells with Aβ deposits characterized by distinct intracellular Aβ/HNE ratios, equal to 0.147 and 0.786 for the population with stronger and less intense immunoreactions for HNE, respectively (Figure 9). The intensity of neuronal Aβ immunoreaction was correlated with that for HNE; the correlation coefficient in the populations with higher and lower Aβ/HNE ratio was 0.77 (p < 0.001) and 0.79 (p < 0.001), respectively.

## Discussion

Oxidative stress has been detected in the brain and in peripheral organs of autistic subjects [11-13,18-21]. Our data link intraneuronal accumulation of N-terminally truncated Aβ in dup(15)/autism and in idiopathic autism to elevated intracellular levels of lipid peroxidation products, the accepted markers of oxidative stress. Analysis of the relationship between deposits of Aβ of various sizes and oxidatively modified lipids in three-color immunofluorescence revealed almost complete co-localization of intracellular Aβ with lipid peroxidation products in autism, dup(15)/autism and control brains. However, the absence of HNE and MDA in the majority of the smallest Aβ immunoreactive granules, apparently representing the earliest steps of Aβ accumulation, suggests that cellular accumulation of Aβ precedes formation of lipid peroxidation products. Hence, Aβ deposits are most likely the source of oxidative stress, rather than oxidative stress being the trigger for Aβ accumulation.

Our present and previous [7] studies revealed higher levels of accumulation of N-truncated Aβ in neurons in individuals with idiopathic autism and with dup(15)/autism, than in controls. Detection of Aβ and its N-terminal truncation in both projects was based on immunohistochemical detection with mAbs 4G8 (17–21 aa of the Aβ sequence [30,32]) but not 6E10 (4–13 aa). Even though these antibodies can recognize the epitopes in full-length APP and in APP fragments [35], in human brains fixed in formalin for at least several months, dehydrated in ethanol and embedded in polyethylene glycol the mAbs 4G8 and 6E10 do not react with APP, but detect only Aβ [7,27,28]. Thus, the deposited Aβ species are most likely mainly Aβ17-40/42 [36], Aβ11-40/42 [36,37], Aβ11(pE)-40/42 [38], and possibly also other.

To evaluate the amounts of Aβ and also lipid peroxidation products we applied in this report measuremets of fluorescence in digital images, instead of morphological evaluation of intensity of the immunoreaction. This new approach—quantification of relative protein amounts basing on immunofluorescence imaging—has been recently shown to be a reliable method [39]. Using known aliquots of cytochrome C embedded in gelatin these authors revealed a high accordance of the amounts of protein detected with primary and secondary antibodies and fluorescence microscopy with the actual protein levels present.

The presence of large deposits of N-truncated Aβ inside neurons suggests that the peptide is, at least in part, aggregated. N-truncation of Aβ peptides is known to enhance their aggregation and formation of the β-sheet structure [40]. The biological effects of accumulation of N-truncated Aβ are not well characterized. The peptides have neurotoxic properties—especially the Aβ 17-42 species [40,41]—leading to apoptosis mediated mainly by the caspase-8 and caspase-3 pathways [41]. However, we did not observe apoptotic nuclei in neurons, possibly because of the young age of the subjects. Aggregated and oligomerized full-length Aβ 1-40/42 is involved in the formation of reactive oxygen species through binding transitional metals copper and iron. N-terminal truncation of Aβ lowers the ability to form reactive oxygen species, because copper is bound to His6, His13 and His14, in the N-terminal sequence of Aβ, whereas the carbonyl of alanine-2 is an oxygen ligand [10]. However, methionine-35 can also be oxidized to form a sulfuranyl radical, which subsequently can cause lipid peroxidation [8,9]. These data and the results presented in this study suggest that enhanced accumulation of intracellular N-truncated Aβ may result in increased production of reactive oxygen species and increased formation of lipid peroxidation products.

Our finding of higher levels of lipid peroxidation products in neurons in autism and dup(15)/autism than in controls is in agreement with the reported significantly increased levels of MDA in lysates of the cerebral cortex and cerebellum of autistic subjects [42]. The localization of lipid peroxidation products in almost all mitochondria, in some autophagic vacuoles and lysosomes, and in all lipofuscin granules most likely reflects the sites of formation of lipid peroxidation products, their intracellular trafficking, and storage of non-degradable components. Mitochondria generate the superoxide anion radical (O_2_*-), hydrogen peroxide (H_2_O_2_), and hydroxyl radical (HO*) in the electron transport chain reactions (review [43]). The increased levels of lipid peroxidation products in mitochondria in idiopathic autism and dup(15)/autism (Figure 2), as well as their co-localization with N-truncated Aβ, may have very significant biological consequences for the neuron. In mitochondria, MDA has an inhibitory effect on mitochondrial complex I- and complex II-linked respiration and significantly elevates production of reactive oxygen species and protein carbonyls [44]. Thus, increased formation of HNE and MDA in neurons with N-truncated Aβ deposits may enhance the formation of reactive oxygen species in mitochondria and may be the cause of, or enhance an existing mitochondrial dysfunction in autism and dup(15)/autism. Impaired mitochondrial function has been detected in children with autism, with increased rates of hydrogen peroxide production [45], and reduced expression of numerous genes of mitochondrial complex I, III, IV, and V [46]. In autism the levels of HNE protein adducts are also increased in erythrocyte membranes and in plasma; the imbalance between pro- and anti-oxidative mechanisms may be linked to higher levels of unbound iron [47].

The presence of lipid peroxidation products in lysosomes and in all lipofuscin granules in neurons reflects the sites of processing of oxidatively modified molecules, which are known to be partially degraded in lysosomes and deposited in lipofuscin [48,49]. Detection of lipofuscin in brain neurons in children with autism, even younger than 10 years of age, is in agreement with the reported previously increased frequency of cells containing lipofuscin—particularly in Brodmann areas 22 and 39—in autism [50].

We have not found significantly increased levels of total HNE- and MDA-modified proteins in whole brain lysates, possibly because the Aβ-accumulating neurons constitute only a minor portion of the brain cortex, or because significant fractions of HNE and MDA detected by immunofluorescence were not protein-bound. However, lipid peroxidation products are known to modify proteins. The binding is aminoacid–specific: for HNE, it is histidine (particularly when flanked by basic amino acid residues), and less frequently Cys, and Lys. Proteins particularly susceptible to modifications by HNE include enzymes involved in glycolysis and ribosomal proteins [51]. Covalent binding of HNE to enzymes frequently causes their quick inactivation e.g., glyceraldehyde-3-phosphate dehydrogenase [52] and ion-transporting ATPases [53,54].

Detection of HNE and MDA in the nuclei of numerous neurons points out to yet another possible biological effect of lipid peroxidation products—their influence on transcription. MDA reacts with DNA forming guanine derivatives, which in the transcribed DNA strand of expressed genes strongly inhibit RNA polymerase II [55]. MDA can also affect transcription through modification of aldehyde dehydrogenase 2—which in the nucleus plays an important role in transcription repression through its interaction with histone deacetylases—by inhibiting its nuclear translocation and its repressive activity in general transcription [56]. The great variability of nuclear reactions for HNE and MDA (Figures 4**,**6, 7, 8, 9) suggests that susceptibility for this effect of lipid peroxidation products may vary among cell subpopulations.

Cellular co-localization of lipid peroxidation products and Aβ may have significant biological consequences. HNE may covalently bind Aβ at multiple locations through 1,4 conjugate addition and/or Schiff base formation, which leads to covalent cross-linking of Aβ and formation of Aβ protofibrils [57]. Furthermore, oxidative stress can even trigger deposition Aβ, as shown for cultured brain vascular smooth muscle cells [28,29]. Accumulation of HNE may also increase neuronal levels and secretion of Aβ by up-regulating expression of BACE-1—APP secretase-β—through activation of the c-Jun N-terminal kinases and p38 [58]. Increased Aβ levels and oxidative stress in neuronal progenitor cells may impair neurogenensis in postnatal brain. Full-length Aβ—even the less neurotoxic species 1-40—causes oxidative damage in human neuronal progenitor cells and impairs proliferation, migration, and formation of processes [59].

Analysis of the levels of Aβ and HNE in individual neurons indicates the existence of two neuronal populations in the frontal cortex with a distinct relationship between accumulation of Aβ and HNE: in one, the ratio is similar to that for Aβ and MDA; in the other, moderate contents of Aβ were accompanied by a massive accumulation of HNE. This significantly enhanced formation of HNE in a subpopulation of neurons could be related to some neuronal lineages and/or to a certain functional state of neuron. Further studies are necessary to characterize cells with these properties.

## Conclusions

We propose that formation of deposits of N-truncated Aβ, which become a source of reactive oxygen species and lipid peroxidation products, causes neuronal dysfunction in autism. Accumulation of lipid peroxidation products causes/increases dysfunction of mitochondria and further increases Aβ accumulation leading to a self-enhancing process. Thus, autism appears to be an Aβ-associated disorder with enhanced non-amyloidogenic processing of APP and abnormal cytoplasmic accumulation and trafficking of N-terminally truncated Aβ, which lead to enhanced oxidative stress and mitochondrial injury, contributing to abnormal neuron development and function.

## Competing interests

The authors declare that they have no competing interests.

## Authors’ contributions

JF: concept and design of the experiments, immunohistochemical and WB studies and measurements, data analysis and interpretation, and writing the manuscript. BMK: design of the experiments, immunohistochemical studies, data analysis and interpretation, and writing the manuscript. NCS: genetic and clinical characterization of duplication 15q11.2-q13 individuals. WTB: study design, clinical and genetic evaluation of the individuals studied, and writing and editing the manuscript. JW: study design, neuropathological and cytoarchitectonical characterization of the brains, writing the manuscript. All authors read and approved the final manuscript.
